# Time trends and area-level socioeconomic inequalities in early childhood development at 2–2.5 years old in England between 2019 and 2025: a local area ecological study

**DOI:** 10.1136/bmjph-2025-003161

**Published:** 2026-08-03

**Authors:** Yu Wei Chua, Caitlin Murray, Luke Munford, Davara Bennett, Dougal Hargreaves, David Taylor-Robinson

**Affiliations:** 1 Department of Public Health, Systems and Policy, University of Liverpool, Liverpool, UK; 2 The University of Manchester, Manchester, UK; 3 Imperial College London School of Public Health, London, UK

**Keywords:** Community Health, COVID-19, Public Health

## Abstract

**Introduction:**

There are gaps in national data on early childhood development to inform progress in closing the inequality gap and to capture the impact of the COVID-19 pandemic on children’s development in different developmental domains. This longitudinal area-level ecological study examined time trends in socioeconomic inequalities in early childhood development at 2–2.5 years in England’s local authorities between 2019 and 2025.

**Methods:**

We included 2 105 384 children from 119 upper-tier local authorities. Rates per 100 children not on track in development at 2–2.5 years, assessed on the Ages and Stages Questionnaire 3, were analysed. Area-level income deprivation was assessed using the Income Deprivation Affecting Children Index 2019, converted to the Slope Index of Inequality (SII) and Relative Index of Inequality (RII). Linear mixed-effect models assessed the fixed effects of year, year^2^ and income deprivation on early childhood development in any developmental domain and in communication, gross motor, fine motor, personal social or problem solving.

**Results:**

The average rate of children not on track in any developmental domain increased from 15.2 (95% CI 13.7 to 16.7) in 2019, peaking in 2023 (19.1 (17.5 to 20.7)), but with little sign of recovery by 2025 (year: 1.09 (1.05 to 1.13); year^2^: 0.99 (0.99 to 1.00)). Rates for communication, personal social skills and problem solving increased more steeply than gross motor and fine motor. The most versus least income-deprived areas had worse developmental outcomes throughout the period (SII: 5.8 (4.7 to 7.0); RII: 44% (35% to 55%)), with no evidence of year x income deprivation interactions. The largest absolute inequality gap was in communication (SII: 4.7 (4.0 to 5.7); RII: 56% (46% to 72%)).

**Discussion:**

In England, early childhood development worsened during the pandemic and children born after the pandemic continue to be affected. Area-level inequalities were striking but did not worsen during this period. Pandemic-recovery efforts need to consider the potentially enormous economic and societal cost of disruption to early childhood development.

**WHAT IS ALREADY KNOWN ON THIS TOPIC**

- The COVID-19 pandemic has impacted early childhood development, but some domains may be particularly affected. Gaps in early childhood developmental data internationally limit understanding of whether children from deprived backgrounds fared worse.

**WHAT THIS STUDY ADDS**

- In England, communication, gross motor, fine motor, problem-solving and personal social skills at 2–2.5 years worsened in the wake of the COVID-19 pandemic, with communication skills most impacted. Stark socioeconomic inequalities were observed throughout the period of 2019–2025. The economic cost of disrupted early childhood development may be huge.

**HOW THIS STUDY MIGHT AFFECT RESEARCH, PRACTICE OR POLICY**

- Policy makers need to prioritise early years and children services in pandemic recovery efforts to improve early childhood development, especially for children from socioeconomically deprived backgrounds.

## Introduction

 Every child has a right to develop to their full potential. Promoting early childhood development is a foundation for all children to reach their full potential and a priority for sustainable development.^1–5^ Yet children experience unfair, unjust and avoidable differences in health and development due to their early life socioeconomic circumstances. These socioeconomic inequalities in health and developmental outcomes, evidenced from conception to adulthood,^6^ stem from differences in the economic, material and psychosocial conditions experienced by different socioeconomic groups.^7^

In high-income countries, the impact of recent social and economic trends on child development is a growing concern, especially the rise in child poverty^8^ and the impact of COVID-19.^9^ In the UK, child poverty has risen since 2010 and around 30% of children currently live in relative poverty.^10^ COVID-19 led to an unprecedented change to children’s social environments, resulting from school closures, social isolation and disruption to social support services.^9^ Inequalities in poverty, school readiness and other health and educational outcomes across childhood were widening in the UK before the pandemic.^11^ These changes to children’s social environments are expected to disrupt children’s development, and children from more disadvantaged socioeconomic backgrounds are likely to be more vulnerable.

Extensive evidence has shown the detrimental impact of socioeconomic disadvantage on early childhood development, but there remain gaps in national and cross-national data and measurement tools to track the current state of early childhood development. Previous evidence includes data applying both individual (eg, parental education and household income) and neighbourhood (eg, index of deprivation, access to services) measures of socioeconomic status.^12,13^ Some evidence is based on national birth cohort studies and large population level analyses.^14,15^ However, these data capture a static picture of children born over a decade ago. Up-to-date, population-level data is needed to track time trends in early childhood development, progress in closing inequalities and provide intelligence for policymakers on the children most in need of early support. Monitoring progress in early developmental outcomes at a population level helps support financial and political commitments to improve these outcomes and monitor the impact of policies, programmes and investment in early childhood.^16^

To inform recovery efforts after the COVID-19 pandemic, it is imperative that we understand the extent to which the pandemic impacted children’s development, including any worsening inequalities in early childhood development. Experts had warned during the COVID-19 pandemic that household economic shocks, impact on caregiver stress and ability to provide nurturing care, and disruption of services can disrupt early childhood development.^17^ Evidence now shows that preschool children and infants born during or exposed to COVID-19 pandemic were delayed in their development, compared with children not exposed to the pandemic.^18–20^ However, some studies have suggested that certain domains may be unaffected and that communication development may be most affected.^21–23^ Evidence of whether preexisting socioeconomic inequalities in early childhood development were exacerbated by the pandemic remains limited and mixed.^24–27^ Gaps in national surveillance data in Europe meant that policy makers failed to capture trends in early childhood development in a timely manner,^28^ hindering an adequate response to the unequal impact of the pandemic on children’s early development.^11^

In the UK, population-level indicators of early childhood development have previously relied on indicators of school readiness at school entry. Recently available data on early childhood development assessed at 2–2.5 years old now enables monitoring of outcomes nationally, just after the critical first 1000 days. Child-level data are submitted by health visitors to the Community Services Dataset, but most of the data is not yet research-ready.^29^ No study has examined the area-level aggregated data available in the interim.^30^ Using area-level estimates, this study assesses national time trends and socioeconomic inequalities in the rates of children not developmentally on track at 2–2.5 years in any five domains of development, and in each domain, in England around the time of the COVID-19 pandemic.

## Methods

### Study design and setting

We report an ecological area-level analysis assessing longitudinal trends in children not developmentally on track at 2–2.5 years between 2018/2019 and 2024/2025 (hereafter 2019–2025) in England, the extent of socioeconomic inequalities and whether inequalities changed during this period. The unit of analysis is at the upper-tier local-authority level, which is the administrative body responsible for governing and delivering major public services (eg, education, social care, transport) in defined areas of the UK.

### Eligibility criteria

Due to the novel nature of the dataset, we carried out preliminary analyses of data quality and coverage for the years 2018–2025 to determine inclusion in the study (see online supplemental 1 and the Statistical methods section). Based on the preliminary analyses, we included local authorities with published data on the rate of children not developmentally on track at 2–2.5 years in the years 2019–2024. Data from Cumberland, Westmorland and Furness were excluded due to irreconcilable local authority boundary changes during this period. Data from local authorities with small population sizes were combined (Isles of Scilly with Cornwall, City of London with Hackney). We excluded the most extreme top 1% of data points representing anomalously high rates of children not on track in any domain (data from Barnet, 2023, Ealing (2023, 2024), Richmond upon Thames (2023) and Brent (2020)). We excluded data from Torbay (2020, 2024) due to rates of zero, which suggests the data collected or entered is potentially of poor quality.

### Data sources and measures

Outcome data from 148 local authorities (after accounting for boundary changes) were obtained from the Health Visiting Service Delivery Metrics, published by the Office of Health Improvement and Disparities.^30^

As part of the universal Healthy Child Programme, health visitors carry out mandatory health and developmental reviews at antenatal contact, and postnatally at the new birth visit, 6–8 weeks of age, 12 months of age and 2–2.5 years of age.^31^ The Ages and Stages Questionnaire 3 (ASQ-3) has been used routinely since 2015 to assess early childhood development at 2–2.5 years. The ASQ-3 is a parent-completed developmental screening tool for children aged 1–66 months in five domains: communication, gross motor, fine motor, problem solving and personal social skills.^32^ Scoring below cut-off scores (greater than 2 SD below the mean, based on US norms) indicates that children do not meet expected development in a specific domain, and therefore the need for further developmental evaluation. Originally developed in the USA, the ASQ-3 has been validated internationally, although population norms are not available for England.^33^ We obtained the rate of children *not* developmentally on track, per 100 children assessed on the ASQ. Data from eight assessment years (2018–2025) were available at the time of analysis.

The percentage of children receiving developmental reviews was over 90% in all years, but lower for children receiving an ASQ-3 review.^34^ As the early childhood developmental metric was classed as experimental statistics up to 2021 due to data quality and coverage of the ASQ review,^35^ we also obtained data on the quality (number of local authorities submitting publishable quality data) and coverage of ASQ-3 review (percentage of eligible children receiving an ASQ-3 review).

Our exposures were the assessment year and childhood socioeconomic conditions. Childhood socioeconomic conditions were defined as area-level income deprivation, measured using the Income Deprivation Affecting Children Index Average Score from the 2019 Indices of Multiple Deprivation, created by the UK Office for National Statistics.^36^ This score measures the proportion of children experiencing income deprivation in the LA, averaged across deprived and non-deprived areas. A higher score indicates greater income deprivation. We ranked local authorities and created quintiles where a fifth of the 2019 child population fell into each income deprivation quintile. A higher quintile indicates greater socioeconomic advantage, in line with literature using the Index of Multiple Deprivation quintiles to examine area-level inequalities.

We used ranks of the continuous score to estimate our primary measures of inequality, the slope and relative index of inequality (SII and RII), regression-based indices of inequality obtained by regressing the health outcome of interest on a relative socioeconomic position (ie, rank). In additive models, the regression coefficient of rank represents the SII, representing the absolute difference in outcome between the theoretical extremes of the socioeconomic hierarchy. In multiplicative models, the regression coefficient represents the ratio between the theoretical extremes of the socioeconomic hierarchy, that is, the relative difference in the outcome. Since we use multiplicative models (see the Statistical methods section), we obtained the RII directly from the regression coefficients and calculated the SII from model predictions of the theoretical most and least advantaged groups. We expected that the least advantaged would have lower rates of children not developmentally on track. Therefore, to avoid double negatives and increase interpretability, we converted the ranks into a continuous score ranging from 0 (most advantaged) to 1 (least advantaged), such that model parameters capture the absolute and relative difference in children not developmentally on track in the least compared with the most advantaged.

We identified covariates using directed acyclic graphs to isolate the relationship of interest and address potential selection bias (online supplemental eFigures 1 and 2). Children of non-white ethnicity were previously found to be less likely and children from the most deprived decile more likely to receive an ASQ review, in 2019 and 2020 data.^26^ Selection bias can occur if these factors, and any unobserved factors affecting review, are also associated with the outcome (see online supplemental 1 for additional details). There was a background trend of generally improving employment rates in households with children.^37^ This trend may hide adverse effects of COVID-19 lockdown on loss of employment. As our focus was on time trends related to COVID-19 impact, we controlled for ethnic composition (percentage of 0–15-year-old children of White British/Irish ethnicity from the 2021 UK Census),^38^ income deprivation and employment rates at birth (Annual Population Survey/Labour Force Survey, households with children 0–15 years, by combined economic activity status^39^). In analyses of income deprivation, we controlled for year and ethnicity. We did not control for ASQ coverage, which may be a confounder, but also a collider. This is because coverage is a proxy of all the factors affecting whether a child receives an ASQ review, and if both the exposure (income deprivation or year) and the outcome (developmental delays) are associated with receiving an ASQ review, this would also bias the results. Since we exclude the most severe influences of ASQ coverage (see next section), and control for the main factors influencing review completion (ethnicity and deprivation), we approached the main analysis without further adjusting for residual variation in coverage.

### Statistical methods

Analyses were carried out in R (V.4.3.2). We preliminarily described data quality and coverage from 2018 to 2025 to inform main analyses (online supplemental eTable 1, eFigures 1 and 2). We excluded data with poor coverage (<75%) based on the preliminary analyses and excluded data from 2018. Due to high positive skewness and kurtosis of the outcome (online supplemental eTable 2), we log-transformed the outcome and checked regression assumptions (online supplemental eFigures 3–7).

In the main analyses, we first obtained means and 95% CIs of each outcome over time and by income deprivation.

Second, in the model building step, we used model fit indices (Akaike Information Criterion (AIC), Bayesian Information Criterion (BIC)) and likelihood ratio tests to determine the random and fixed effects structure. We followed a step-down procedure starting with the most complex model, including random intercepts of local authority, fixed and random slopes of linear and quadratic year, and fixed effects of income deprivation and interactions with linear and quadratic year. The best fit models included random intercepts and random linear slopes for year for all outcome domains (online supplemental eTable 3), allowing heterogeneity across local authorities in the time trends. Linear and quadratic year fixed effects for any domain, communication and personal social skills, and linear year for gross motor, fine motor and problem solving (online supplemental eTable 4). There was no evidence of interaction effects (online supplemental eTable 4).

Third, we estimated the effects of year and income deprivation, controlling for ethnic composition. Although we also considered employment rates at birth as a confounder of year effects, this did not change model parameters (online supplemental eTable 5).

Fourth, we visualised longitudinal trends using predicted means and CI at each year and calculated the differences relative to 2019. We obtained the RII by exponentiating the coefficient for income deprivation, estimated on the log-scale. For the SII, we obtained exponentiated predictions for the least advantaged versus the most advantaged, and calculated the difference in predicted values on the original scale. 95% CIs were generated using 1000 non-parametric bootstrap samples of the original data.

Finally, using the absolute difference in the rate of children not on track in any developmental domain between 2019 and 2025, we use population estimates and cost estimates in the published literature to estimate potential contemporaneous and long-term implications of the increase in children not on track in development, on service use and economic losses (online supplemental eMethods). We focus on key domains of speech and language therapy, special educational needs, parental employment losses and projected implications on workforce participation and labour productivity.^40–42^

### Sensitivity analyses

To test if findings were robust despite excluding substantial data with poor ASQ coverage, we repeated the analysis using data with a more lenient criterion for coverage (defined as at least 50% coverage), including adjustment for ASQ coverage to control for the influence of data quality on the outcome (see online supplemental eMethods).

## Results

### Sample characteristics

In analyses of data quality and coverage from 2018 to 2025, availability of publishable quality data was poor in 2018, but later years had comparable levels of data: 74 (50.0%) local authorities submitted publishable ASQ data in 2018, 113 (76.4%) in 2019 and rising to 139 (93.9%) in 2025, with some fluctuations annually (online supplemental eTable 6). Income deprivation score was correlated with ASQ coverage in 2018 (τ=0.141, p=0.015), 2020 (τ=0.126, p=0.029) and 2021 (τ=0.120, p=0.034), the first year of data publication and during the pandemic, but not in other years (online supplemental eTable 7). This indicates that coverage was better in more income-deprived areas, suggesting targeting of ASQ reviews to disadvantaged groups. Most local authorities achieved over 50% ASQ coverage, but there was variability in coverage, and very low coverage in some local authorities or years (online supplemental eTable 8). We proceeded with analysing data from 2019 onwards, with at least 75% ASQ coverage (n=119 (80.4%) local authorities) (online supplemental eFigure 8; see online supplemental eTable 9 for a breakdown of the number of local authorities included in each year). Out of 2 105 658 children eligible for a 2–2.5-year developmental review from 2019 to 2025, 1 796 686 (85.3%) children were assessed on the ASQ and therefore included in the area-level rates analysed.

**Figure 1:**
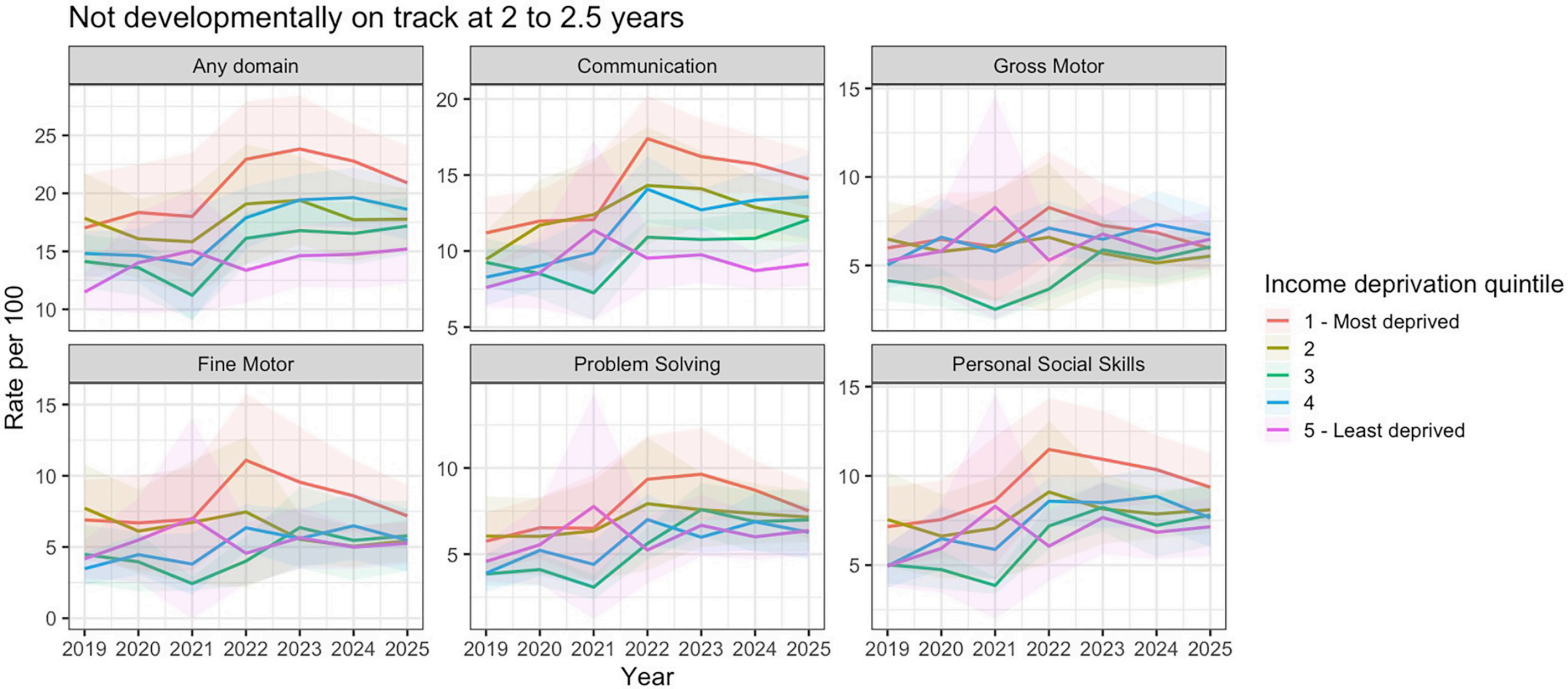
Annual rates of children not developmentally on track at 2–2.5 years in English local authorities—means with 95% CIs by quintiles of income deprivation.

Rates per 100 children not on track in any domain increased from 15.2 (95% CI 13.7 to 22.5) in 2019, peaking in 2023 at 19.1 (17.5 to 20.7) and decreasing slightly to 17.9 (16.6 to 19.1) in 2025 (table 1). On average between 2019 and 2025, the rate of children not on track in any domain was higher in local authorities in the least advantaged quintile (rate per 100: 20.9 (19.3 to 22.5)) compared with most advantaged quintile (14.2 (12.9 to 15.4)), but there was a high degree of overlap in the CIs for quintiles 2, 3, 4 and 5 (table 1, figure 1). Rates were highest across time for communication. The greatest absolute difference comparing the most and least advantaged was also in communication (table 1).

**Table 1:** Descriptive statistics of the numbers and rates (per 100 assessed) of children not developmentally on track

	Any domain	Communication	Gross motor	Fine motor	Problem solving	Personal social skills
Number of children assessed 2019–2025	1 794 281	1 797 167	1 797 448	1 797 845	1 797 738	1 797 739
Number of children not developmentally on track 2019–2025	314 629	206 051	111 198	110 489	114 409	137 398
Rate of children not on track in each year (mean (95% CI))						
2019	15.2 (13.7 to 16.7)	9.2 (8.3 to 10)	5.5 (4.7 to 6.3)	5.5 (4.4 to 6.6)	4.9 (4.2 to 5.7)	6.1 (5.1 to 7)
2020	15.5 (13.9 to 17.1)	10.1 (9.1 to 11.1)	5.7 (4.7 to 6.8)	5.4 (4.2 to 6.6)	5.6 (4.7 to 6.4)	6.4 (5.4 to 7.3)
2021	15.2 (12.9 to 17.4)	10.9 (9.2 to 12.7)	5.8 (4.2 to 7.3)	5.7 (3.8 to 7.6)	5.8 (4.3 to 7.3)	6.9 (5.3 to 8.5)
2022	18 (16.2 to 19.9)	13.4 (12.1 to 14.7)	6.3 (5.1 to 7.5)	6.8 (5.2 to 8.4)	7.1 (6 to 8.2)	8.6 (7.4 to 9.7)
2023	19.1 (17.5 to 20.7)	12.9 (11.9 to 13.9)	6.5 (5.6 to 7.3)	6.6 (5.4 to 7.9)	7.5 (6.6 to 8.4)	8.8 (7.9 to 9.7)
2024	18.6 (17.1 to 20.1)	12.5 (11.6 to 13.5)	6.2 (5.4 to 6.9)	6.3 (5.3 to 7.3)	7.3 (6.5 to 8)	8.4 (7.6 to 9.2)
2025	17.9 (16.6 to 19.1)	12.2 (11.3 to 13.2)	6.1 (5.5 to 6.8)	5.8 (4.9 to 6.7)	6.9 (6.2 to 7.6)	8 (7.3 to 8.7)
Rate of children not developmentally on track by income deprivation quintile (mean (95% CI))						
1—least advantaged	20.9 (19.3 to 22.5)	14.4 (13.4 to 15.3)	6.7 (5.9 to 7.5)	8.2 (6.9 to 9.4)	7.8 (7 to 8.6)	9.4 (8.5 to 10.4)
2	17.6 (16.2 to 19)	12.3 (11.3 to 13.3)	5.9 (5 to 6.7)	6.2 (5.1 to 7.3)	6.9 (6 to 7.8)	7.8 (6.9 to 8.6)
3	15.4 (14.4 to 16.4)	10.2 (9.5 to 10.9)	4.7 (4.1 to 5.2)	4.8 (3.9 to 5.7)	5.7 (5.1 to 6.3)	6.5 (5.9 to 7.1)
4	17.4 (16.4 to 18.5)	11.8 (11 to 12.7)	6.5 (5.9 to 7.2)	5.3 (4.5 to 6)	5.8 (5.3 to 6.4)	7.5 (6.8 to 8.1)
5—most advantaged	14.2 (12.9 to 15.4)	9.1 (8.3 to 9.8)	6.1 (5.3 to 6.9)	5.2 (4.3 to 6.1)	6 (5.2 to 6.7)	6.7 (5.8 to 7.5)

### Time trends in early childhood development

**Figure 2:**
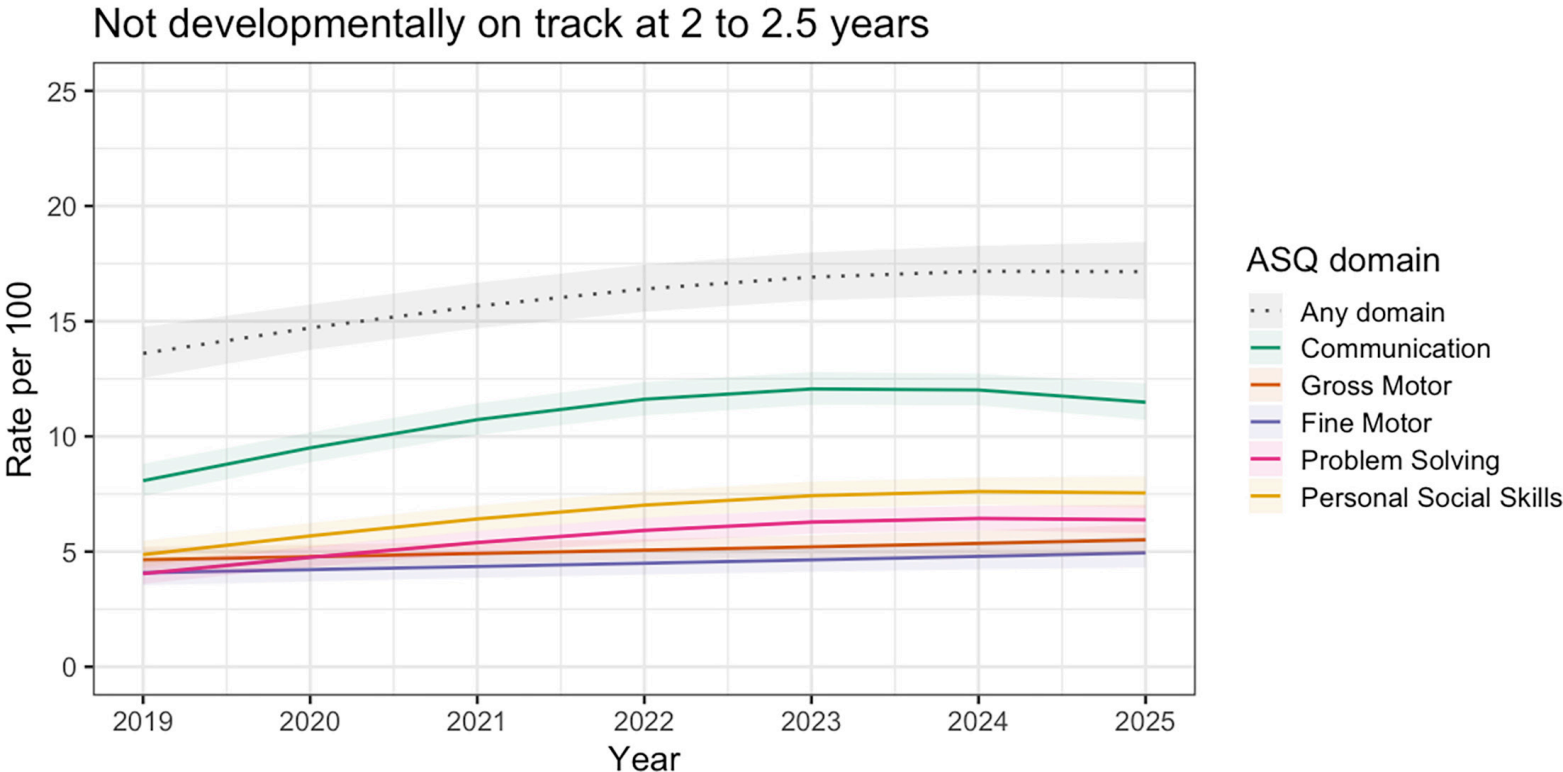
Predicted mean and CIs of children not developmentally on track in any domain and each domain over 2019–2025. ASQ, Ages and Stages Questionnaire.

In mixed effect models adjusted for income deprivation and ethnicity, there was evidence for a non-linear, quadratic trend of year for children not on track in any domain (year: 1.09 (1.05 to 1.13); year^2^: 0.99 (0.99 to 1.00)). The small quadratic effect of year indicates that rates were largely rising over this period with little sign of recovery by 2024, the year in which children assessed did not experience the pandemic lockdown (table 2, figure 2). Compared with 2019 rates, in 2025 the rate of children not on track in any domain had risen by 3.6 (2.4 to 4.5) per 100, or a relative increase of 26% (1.26 (1.17 to 1.34)) (online supplemental eTable 10). Adjusted and unadjusted estimates were the same.

**Table 2:** Mixed effect models of year and income deprivation, controlled for per cent white ethnicity

Predictors	Any domain	Communication	Gross motor	Fine motor	Problem solving	Personal social skills
	Estimates	Estimates	Estimates	Estimates	Estimates	Estimates
(Intercept)	13.82***	8.90***	3.49***	3.34***	3.46***	4.45***
	(10.32 to 18.50)	(6.74 to 11.76)	(2.20 to 5.54)	(1.87 to 5.96)	(2.33 to 5.13)	(3.05 to 6.48)
Year	1.09***	1.20***	1.03*	1.03*	1.19***	1.19***
	(1.05 to 1.13)	(1.15 to 1.26)	(1.01 to 1.05)	(1.00 to 1.06)	(1.12 to 1.27)	(1.12 to 1.25)
Year^2^	0.99**	0.98***			0.98***	0.98***
	(0.99 to 1.00)	(0.97 to 0.99)			(0.97 to 0.99)	(0.98 to 0.99)
Income deprivation	1.44***	1.56***	1.13	1.74**	1.47**	1.49**
	(1.18 to 1.76)	(1.30 to 1.88)	(0.82 to 1.55)	(1.17 to 2.60)	(1.13 to 1.93)	(1.16 to 1.91)
white ethnicity per cent	1.00	1.00**	1.00	1.00	1.00	1.00
	(0.99 to 1.00)	(0.99 to 1.00)	(1.00 to 1.01)	(0.99 to 1.00)	(1.00 to 1.00)	(0.99 to 1.00)
Random effects						
σ^2^	0.04	0.06	0.14	0.18	0.13	0.09
τ_00_	0.12_AreaName_	0.12_AreaName_	0.25_AreaName_	0.42_AreaName_	0.21_AreaName_	0.24_AreaName_
τ_11_	0.00_AreaName.I(YearN)_	0.00_AreaName.I(YearN)_	0.01_AreaName.I(YearN)_	0.01_AreaName.I(YearN)_	0.00_AreaName.I(YearN)_	0.01_AreaName.I(YearN)_
ρ_01_	−0.53_AreaName_	−0.66_AreaName_	−0.44_AreaName_	−0.45_AreaName_	−0.57_AreaName_	−0.68_AreaName_
ICC	0.71	0.56	0.62	0.68	0.56	0.63
N	118_AreaName_	118_AreaName_	118_AreaName_	118_AreaName_	118_AreaName_	118_AreaName_
Observations	531	533	533	533	533	533
Marginal R^2^/conditional R^2^	0.138/0.754	0.233/0.662	0.016/0.628	0.055/0.698	0.124/0.615	0.135/0.679
AIC	210.82	364.69	804.28	963.96	706.80	613.09

AIC, Akaike Information Criterion; ICC, intraclass correlation coefficient.

Rates of children not on track in communication, personal social skills and problem solving increased most steeply, stabilising and decreasing slightly as indicated by a small quadratic effect (table 2, figure 2). Rates of children not on track in gross motor and fine motor increased linearly, with a gentler gradient (table 2, figure 2). Communication showed the greatest absolute increase by 2023, followed by similar increases in personal social skills and problem solving, and smaller increases in fine motor and gross motor (figure 2, online supplemental eTable 10).

### Socioeconomic inequalities in early childhood development

The income deprivation coefficient in mixed effect models (adjusted for year and ethnicity) (1.44 (1.18 to 1.76)) was slightly smaller than unadjusted estimates (1.49 (1.22 to 1.81)). Using bootstrapped predictions from the adjusted income deprivation coefficient, we found evidence of absolute inequalities (SII: 5.8 (4.7 to 7.0) per 100) and relative inequalities (RII: 1.44 (1.35 to 1.55)) in rates of children not on track in any domain (table 3). This means that the least advantaged areas had 5.8 per 100 greater or a 44% greater rate of children not on track in any domain. The lack of evidence of interaction effects between income deprivation and year indicates that the extent of area-level inequality was stable over this period.

**Table 3:** Estimates (mean (95% CI)) of the slope and relative index of inequality based on the income deprivation model coefficient, using 1000 bootstrapped samples

	Any domain	Communication	Gross motor	Fine motor	Problem solving	Personal social skills
Slope index of inequality	5.8 (4.7 to 7.0)	4.7 (4.0 to 5.7)	0.6 (0 to 1.3)	2.5 (1.8 to 3.2)	2.1 (1.5 to 2.9)	2.6 (2.0 to 3.3)
Relative index of inequality	1.44 (1.35 to 1.55)	1.56 (1.46 to 1.72)	1.13 (1 to 1.31)	1.74 (1.5 to 2.06)	1.49 (1.32 to 1.71)	1.49 (1.35 to 1.67)

We found inequalities in all developmental domains, except gross motor. The largest absolute inequality was in communication (SII: 4.7 (4.0 to 5.7)), but relative inequalities were similar across affected developmental domains (range: 1.49–1.74) (table 3).

### Sensitivity analyses

In sensitivity analyses, we repeated models on a larger sample of data (143 (96.6%) local authorities, 2 538 933 (78.4%) children assessed on the ASQ out of 3 237 567 eligible children) based on more lenient inclusion criteria for coverage (at least 50%). In models with adjustment for coverage to account for the influence of poor coverage on rates of children not on track, parameter estimates for the year were the same. The main analyses which excluded more data with poorer quality may have overestimated the relative inequality in children not on track in any domain and communication and underestimated the inequality in fine motor, problem solving and personal social skills, with a 5%–7% relative difference in the parameter estimates (online supplemental eTable 11). However, the CIs for parameter estimates of income deprivation mostly overlapped.

### Service use and economic costs

Compared with 2019, in 2025, an approximate 4 per 100 greater rate of children not on track in any domain could mean 21 600 more children require child health services nationally, using a population estimate of approximately 540 000 children eligible for their 2–2.5-year review in 2025. Worse early childhood developmental outcomes in 2025 alone could incur an additional £60 million annually in early intervention and educational costs, and parental employment losses, and potentially enormous costs due to reduced workforce participation or economic productivity in adulthood (online supplemental eTable 12).

## Discussion

Drawing on English national surveillance data, our study provides timely public health intelligence on recent trends and socioeconomic inequalities in early childhood development, as early as 2–2.5 years. Cohorts assessed during or after the pandemic had greater rates of children not developmentally on track in any domain at 2–2.5 years, compared with the 2019, the unexposed cohort. Notably, children born after the pandemic (assessed in 2024 and 2025) continued to experience worse outcomes compared with the unexposed cohort. All developmental domains were impacted by the pandemic, with communication, personal social skills and problem solving more strongly impacted than motor domains. There were stark area-level socioeconomic inequalities which did not change during this period, observed in all domains except gross motor.

### Limitations and future directions

Common limitations in the use of area-level data apply. This includes the modifiable unit area problem, which restricts our findings to spatially aggregated data at the upper-tier local authority level. We expect, based on previous work analysing inequalities at different spatial levels, that inequalities are underestimated when data is aggregated over larger areas.^43^ Furthermore, studies using individual-level data have found a stronger adverse impact of the pandemic on various domains of development among children from low versus high socioeconomic status.^22,25,27,44^ Overall, this suggests our findings are unlikely to result from ecological fallacy, but the extent of inequality may be underestimated at an individual level and area-level data may not be able to detect changes in inequalities. Individual-level data will be best placed to investigate whether the impact of early years exposure to the COVID-19 pandemic may be sustained longitudinally into school age. The novelty of the early childhood development dataset meant that data coverage and quality were poorer, mainly in the first 2 years of the publication. While dealing with these limitations in the selection criteria, greater political and service level commitment to monitoring and reporting can improve future national datasets at both individual and area levels.

### Comparisons with the literature

Our findings, showing that cohorts in England exposed to COVID-19 experienced worse early childhood developmental outcomes in almost all domains, are consistent with large national studies assessing early childhood development at around 2 years of age.^45,46^ Our findings support previous work showing that children born during the pandemic, who experienced a longer exposure to the pandemic, experienced the worst outcomes.^46^ However, unexposed children continue to experience poorer outcomes compared with pre-pandemic unexposed children, suggesting exposure to the pandemic lockdown is not the only driver of poorer early childhood development since the pandemic. Previous work has also found socioeconomic inequalities in early childhood development in all domains before and during the pandemic.^24,25,46^ The key drivers of socioeconomic inequalities before the pandemic include the home learning environment, pressures on the early childhood and care sector, and stress in the home, which are all likely to be exacerbated by the pandemic.^11,47^

Differences in methodology and sample may explain differences between our findings and the wider literature. A relatively small study comparing COVID-19 exposed cohort of 2-year-olds to a historical cohort only found differences in the communication domain.^21^ In studies examining early childhood development in children younger than 1.5 years, or in children of various ages over 0–5, experience of COVID-19 on worse development has been inconsistently reported.^18,19,21,23,46,48–50^ Age of assessment and age of exposure to COVID-19 may explain differences in domain-level impact. Another study reported gross motor delays in younger children exposed to the pandemic, but not in older children,^46^ while exposure to COVID-19 around 2 years old affected only the communication and social interaction domain assessed a year later.^22^ Communication may be the most susceptible, as its development relies on the social environment which was severely restricted by the pandemic. This likely explains why communication delays were the most consistently observed across studies, despite methodological differences.

### Implications for policy and practice

The cost of COVID-19 on inequalities in children’s early development is likely to be enormous and far-reaching. Implications on the economy, society and health services require immediate attention in pandemic recovery efforts. In 2025 alone compared with pre-pandemic, 21 600 more children required further paediatric assessment, adding to already alarming waitlists.^51,52^ The extent of national inequality (5.8 per 100 absolute difference between the worst and least deprived areas) is 1.5 times greater than the adverse developmental impact of the pandemic. An unequal start in life could hinder children from reaching their developmental potential. Spending on late intervention services has risen significantly in the UK post-pandemic, but only small rises have been seen in early intervention spending, after a decade of cuts to early intervention spending.^53^ Our findings emphasise the need for immediate investment in early intervention to address the impact of the pandemic, which could include an additional £4.3 m annually in speech and language therapy services, and an additional £21.6 m annually in the education sector to support the increase in children with special educational needs. Preventative early intervention can support children to reach their full potential, reduce more costly late intervention services and yield long-term economic benefits through improved workforce participation, higher tax revenues, as well as reduced public expenditure on welfare and criminal justice services.

The wide pre-existing socioeconomic inequalities in early childhood development prior to the pandemic, alongside worse early childhood developmental outcomes since the pandemic, do not bode well for the UK government’s school readiness targets. Children from socioeconomically disadvantaged backgrounds are the most at risk of falling behind their peers at school entry. Meeting school readiness targets will require a strong policy focus on inequality reduction, including addressing child poverty, inequalities in the home learning environment and access to early childhood education. The early childhood education and care sector is considered a key sector to alleviate inequalities in early childhood development.^47^ Efforts to tackle the post-pandemic challenges faced by the sector, such as reduced attendance and workforce issues, are urgently needed.^54^

## Conclusions

To conclude, we report stark socioeconomic inequalities and worsening early childhood developmental outcomes at 2–2.5 years in England, during and after the pandemic. All domains were affected, with the most pronounced impact on communicative development. The cost of the pandemic’s impact on early childhood development is likely to be enormous. Ensuring children have a fair start in life requires investment in the early years. National early childhood developmental data can enable monitoring outcomes post-pandemic and progress in closing inequality gaps.

## Supplementary material

10.1136/bmjph-2025-003161 online supplemental file 1

## Data Availability

Data are available in a public, open access repository.
