# Supplementary material for: Time trends and area-level socioeconomic inequalities in early childhood development at 2–2.5 years old in England between 2019 and 2025: a local area ecological study
Source: BMJ Public Health. 2026 Aug 3;4(3):e003161. doi: 10.1136/bmjph-2025-003161 (PMC13475141; doi:10.1136/bmjph-2025-003161)
Supplement: Supplementary file 1 [file bmjph-4-3-s001.docx]

**Time trends and area-level socioeconomic inequalities in early childhood development at 2 to 2.5 years old in England between 2019 and 2025**

Yu Wei Chua, Caitlin Murray, Luke Munford, Davara Bennett, Dougal Hargreaves, David Taylor-Robinson

Corresponding author: Yu Wei Chua ([yu.wei.chua@liverpool.ac.uk](mailto:yu.wei.chua@liverpool.ac.uk))

**Online supplement**

**Supplement 1. Supplemental methods**

**Supplement 2. eTables 1 to 12 and eFigures 1 to 8**

**Supplement 1. Supplemental methods**

***Excluded data due to local authority boundaries***

Data from Cumberland and Westmorland and Furness were excluded, as only available from 2023/24 due to new local authority boundaries and there was no data on IMD 2019. Data from City of London was combined with Hackney. Data from Isles of Scilly was combined with Cornwall.

***Data excluded based on preliminary analyses***

In preliminary analyses, we described data quality and coverage over time in all published data. We obtained the numbers and percentage of local authorities submitting information on the ASQ, distribution in coverage of mandated contacts, and correlation using Kendall rank correlation coefficient between ASQ coverage and IDACI Average Scores within each year. We obtained sample statistics (e.g. mean, standard deviation, skewness, kurtosis) and visualised the spread of data using boxplots, histograms and q-q plots of each early child developmental outcome over all local authorities and years. For all analyses with IDACI Average Scores, data for West Northamptonshire and North Northamptonshire were combined to provide data for Northamptonshire, reflecting local authority boundaries in 2019.

We examined the most extreme 1% of data points with the highest rate of children not developmentally on track in any domain. For example, the highest three rates were 95.8 per 100 children, 89.1 per 100 children and 77.1 per 100 children. We excluded data from Barnet (2023), Ealing (2023, 2024), Richmond upon Thames (2023), Stoke-on-Trent (2018) and Brent (2020). We decided to exclude 2023 data for Barnet (over 800 per 1000 children did not achieve a good level of development in each of the five domain, an anomaly for that year), 2023 and 2024 data from Ealing (Over 75 per 100 children did not achieve a good level of development in communication, compared to less than 10 per 100 children in all other domains, and anomalous from previous years), 2023 data for Richmond-Upon Thames (over 60 per 100 children did not achieve a good level of development in communication compared to less than 50 per children in other domains, anomalous from all other years), 2018 for Stoke-on-Trent and 2020 for Brent (anomalously high rates of children who did not achieve a good level of development in first year of available data, compared to all the other years).

On the lower extreme, we excluded data from Torbay in 2020 and 2024, due to rates of 0 for personal social skills.

We tabulated the number of LAs with outliers above the upper IQR limit (see eTable1). Most of these outliers were excluded once excluding data based on less than 75% coverage.

***Directed Acyclic Graphs***

We were interested in using Year to proxy the effect of COVID-19 shocks. To analyse the effect of Year on the outcome, we controlled for factors affecting review (biasing pathway) by conditioning percent white ethnicity, income d eprivation. We also controlled for improving employment trends in general, that was not resulting from COVID-19 shocks. However, this did not affect parameter estimates of Year, we presented the same model in analyses of Year and Income deprivation (see next paragraph), which includes only percent white ethnicity as a covariate.

Figure 2. Directed Acyclic Graph showing the relationship of interest: Year on the rate of children not developmentally on track.

To analyse the effect of Income deprivation on the outcome, we controlled for Year and percent White ethnicity as confounders. Other factors affecting review could also confound the relationship, but the key predictors - percent white ethnicity and income deprivation were included in the model. We did not control for employment rates as were interested in the full extent which income deprivation affects the outcome, including when income deprivation occurs in the context of unemployment.

Figure 2. Directed Acyclic Graph showing the relationship of interest: income deprivation on the rate of children not developmentally on track.

***Bias due to poor ASQ coverage***

Children of particular characteristics (e.g. non-white ethnicity are less likely to be reviewed). In addition, these characteristics may be associated with poorer early childhood outcomes. This leads to selection bias as we are only able to analyse the outcome amongst children who have been reviewed. As such, amongst local authorities with lower coverage, we expect early childhood developmental outcomes to be better if missing children have poorer outcomes, and outcomes to be poorer if the missing children have better outcomes.

Limiting the analysis only to local authorities with non-missing data and high coverage, however, may also lead to selection bias. This could result in excluding local authorities with high deprivation or a higher percentage of non-white ethnicity – factors which are potentially associated with lower coverage, and which are associated with poorer outcomes.

Most local authorities have ASQ coverage above 50% (eTable 8 and eFigure 2) but this means that half of children may have missed out on a developmental review. As such we decided to analyse data in local authorities with adequate coverage, defined as 75% or greater coverage to address potential bias due to variation in coverage. We did not further control for ASQ coverage because coverage could act as a collider if both the exposure and outcome are associated with it. This is likely since coverage varies by year, and income deprivation was correlated with coverage in 2019. Children who are developmentally delayed may also be more likely to complete an ASQ-3 assessment. In sensitivity analyses, we included data based on at least 50% ASQ coverage, but included adjustment for coverage.

***Model equations***

Let:

${Dev}_{it}$: Rate of children who do not achieve a good level of development, expressed in rate per 1000

${Year}_{t}$: continuous variable centred to 2018/19 as the reference year (range: 2018/19 to 2023/24)

${IncDep}_{i}$: Population-weighted rank (scaled to 0 to 1) of Index of Multiple Deprivation 2019, Income Deprivation Affecting Children Index for each *i* representing *i-th* local authority

${Emp}_{it}$: Percentage of households with children aged 0 to 15 years who are employed in each year, for each *i* representing *i-th* local authority (considered only in models of Year)

${Ethnicity}_{i}$: Percentage of children 0 to 15 years of White ethnicity based on 2021 census, for each *i* representing *i-th* local authority

$\gamma_{0i}$: amount of variation in $\beta_{0i}$ for the *i-th* local authority, assumed to follow a normal distribution ${N(0,\sigma}^{2})$

$\gamma_{1i}$: amount of variation in $\beta_{1i}$ for the *i-th* local authority, assumed to follow a normal distribution ${N(0,\sigma}^{2})$

$\varepsilon_{it}$: residual error, assumed to follow a normal distribution ${N(0,\sigma}^{2})$

Model equation for the effect of Year

$log({Dev}_{it})= \beta_{0i}+\beta_{1i}{Year}_{t}$ $+\beta_{2}{{Year}^{2}}_{t}$ + $\beta_{3}{IncDep}_{i}+{\beta_{4}Ethnicity}_{i}+ {\beta_{5}Emp}_{it}{+ \gamma}_{0i}{+ \gamma}_{1i}Year+\varepsilon_{it}$

Model equation for the effect of income deprivation

$log({Dev}_{it})= \beta_{0i}+\beta_{1i}{Year}_{t}$ $+\beta_{2}{{Year}^{2}}_{t}$ + $\beta_{3}{IncDep}_{i}+{\beta_{4}Ethnicity}_{i}{+ \gamma}_{0i}{+ \gamma}_{1i}Year+\varepsilon_{it}$

***Service-use and economic costs***

We use population estimates of approximately 540,000 children eligible for their 2 to 2.5 year old review in 2025 to calculate the increase in number of children requiring further paediatric assessment.^1^ We assumed that all children falling behind based on the ASQ-3 developmental screen require further paediatric assessment. For both contemporaneous and future costs, we make a conservative assumption that 10% of children failing the screen at 2 to 2.5 years require early interventions and may experience sustained long-term consequences. In other words, we assume that 90% of the additional 4 per 100 children at some point ‘recover’ to the pre-COVID-19 trajectories.^2,3^ Table S2 summarises how the costs were calculated.

For contemporary costs, we expect that some children experiencing developmental delays require early intervention (we focus on speech and language therapy since communication delays are the most prevalent), special educational needs support when they enter school, and additional parental support which may result in parents reducing employment to fulfil childcare duties.

Longitudinal studies in the UK have shown that children showing early developmental delays are less likely to achieve five GCSEs at grade C and above.^4^ Further, recent UK Department for Education estimates that failing to achieve this educational threshold is associated with £96,000 in lifetime earnings.^5^ Based on this estimates, we consider future costs on workforce participation resulting from lower educational attainment. In addition, the Penn Wharton Budget Model (2021) suggests that investments in early childhood development yield long-term economic returns through increased productivity and tax revenue.^6^ Therefore, potential cascading effects of developmental delays on adulthood workforce productivity and affect unemployment rates, may contribute to economic losses.

**References**

1.	Office for National Statistics. Population estimates - local authority based by single year of age - Nomis - Official Census and Labour Market Statistics. Published online 2023. Accessed November 14, 2024. https://www.nomisweb.co.uk/datasets/pestsyoala

2.	Dale PS, McMillan AJ, Hayiou-Thomas ME, Plomin R. Illusory Recovery: Are Recovered Children With Early Language Delay at Continuing Elevated Risk? *Am J Speech Lang Pathol*. 2014;23(3):437-447. doi:10.1044/2014_AJSLP-13-0116

3.	Cleveland Clinic. *Developmental Delay in Children.* 2023. Accessed March 14, 2025. https://my.clevelandclinic.org/health/diseases/14814-developmental-delay-in-children.

4.	Taggart B, Sylva K, Melhuish E, Sammons P, Siraj I. Effective pre-school, primary and secondary education project (EPPSE 3-16+). Published online 2015. https://assets.publishing.service.gov.uk/media/5a803cb240f0b62305b89fbf/RB455_Effective_pre-school_primary_and_secondary_education_project.pdf.pdf.

5.	Hodge L, Little A, Weldon M. *GCSE Attainment and Lifetime Earnings*. 2021. https://assets.publishing.service.gov.uk/media/60c36f0cd3bf7f4bd11a2326/GCSE_Attainment_and_Lifetime_Earnings_PDF3A.pdf

6.	Penn Wharton. *Economic Effects of Preschool and Childcare Programs*. University of Pennsylvania; 2021. https://budgetmodel.wharton.upenn.edu/issues/2021/8/23/economic-effects-preschool-and-childcare-programs

7.	NHS England. *2021/22 National Cost Collection Data Publication.* 2023. https://www.england.nhs.uk/publication/2021-22-national-cost-collection-data-publication/

8.	Hutchinson J. *Identifying Pupils with Special Educational Needs and Disabilities*. Education Policy Institute; 2021. https://epi.org.uk/wp-content/uploads/2021/03/SEND-Indentification_2021-EPI.pdf

9.	OECD. *The Labour Market Position of Families (LMF)*. 2021. https://www.oecd.org/en/data/datasets/oecd-family-database.html#12345.

10.	World Bank Open Data. World Bank Open Data. Accessed March 14, 2025. https://data.worldbank.org

**Supplement 2. eTables 1 to 12 and eFigures 1 to 8**

eTable 1. Number of local authorities with outliers in rates of children not on track in any domain based on boxplots (Rate above 3 times the IQR from the upper IQR limit) by year, for all published data and analysed data

|  | **Outliers in rates of children not on track in Any Domain** | |
| --- | --- | --- |
| **Year** | **All published data** | **Analysed data (excluding coverage <75% and poor quality data** |
| 2018 | 6 | 0 |
| 2019 | 2 | 0 |
| 2020 | 5 | 1 |
| 2021 | 13 | 2 |
| 2022 | 8 | 2 |
| 2023 | 14 | 1 |
| 2024 | 4 | 0 |
| 2025 | 2 | 0 |

eTable 2. Descriptive statistics of ECD outcomes, all published data on children not on track, 2018 to 2025

| **Sample statistics** | **All five domains** | **Communication** | **Gross Motor** | **Fine Motor** | **Problem Solving** | **Personal Social Skills** |
| --- | --- | --- | --- | --- | --- | --- |
| **n** | 996 | 999 | 997 | 997 | 996 | 990 |
| **mean** | 19.0 | 13.4 | 7.1 | 7.2 | 7.7 | 8.8 |
| **sd** | 10.0 | 8.9 | 7.0 | 7.7 | 7.1 | 7.5 |
| **se** | 0.3 | 0.3 | 0.2 | 0.2 | 0.2 | 0.2 |
| **median** | 16.3 | 11.0 | 5.1 | 4.5 | 5.8 | 6.8 |
| **IQR** | 9.7 | 7.0 | 4.7 | 5.8 | 4.6 | 5.4 |
| **min** | 2.5 | 0.4 | 0.2 | 0.2 | 0.5 | 0.0 |
| **max** | 95.9 | 88.0 | 86.7 | 86.2 | 88.7 | 93.9 |
| **skew** | 2.3 | 3.5 | 4.5 | 3.6 | 4.4 | 4.6 |
| **kurtosis** | 9.2 | 18.6 | 32.6 | 21.1 | 30.9 | 35.3 |

eTable 3. Model fit indices of models with random intercepts or random intercepts and slopes, with the best fitting model in bold

| **ASQ domain** | **Random effect terms** | **df** | **AIC** | **BIC** | **ICC** | **Likelihood ratio test (Intercept + linear slope vs Intercept only)** | |
| --- | --- | --- | --- | --- | --- | --- | --- |
|  |  |  |  |  |  | $\boldsymbol{\chi}^{\boldsymbol{2}}$ | **p** |
| Any domain | Intercept | 8 | 424.15 | 460.07 | 0.61 | 24.26 | <0.001 |
|  | **Intercept and linear slope** | 10 | 403.89 | 448.8 | 0.66 |  |  |
| Communication | Intercept | 8 | 620.2 | 656.16 | 0.52 | 13.94 | <0.001 |
|  | **Intercept and linear slope** | 10 | 610.26 | 655.21 | 0.57 |  |  |
| Gross Motor | Intercept | 8 | 1142.63 | 1178.59 | 0.57 | 18.08 | <0.001 |
|  | **Intercept and linear slope** | 10 | 1128.55 | 1173.5 | 0.64 |  |  |
| Fine motor | Intercept | 8 | 1353.76 | 1389.73 | 0.59 | 18.15 | <0.001 |
|  | **Intercept and linear slope** | 10 | 1339.62 | 1384.57 | 0.65 |  |  |
| Problem solving | Intercept | 8 | 972.55 | 1008.5 | 0.53 | 8.42 | 0.004 |
|  | **Intercept and linear slope** | 10 | 968.13 | 1013.06 | 0.57 |  |  |
| Personal social skills | Intercept | 8 | 881.94 | 917.9 | 0.55 | 21.94 | <0.001 |
|  | **Intercept and linear slope** | 10 | 864 | 908.95 | 0.61 |  |  |

*Note 1: All models were fitted with fixed effects of Year + Year^2^ + IncDep + Year*IncDep + Year^2^*IncDep
Note 2: Models fitted with random intercepts, linear and quadratic slopes did not converge*

eTable 4. Model fit indices for Models 1 to 4 comparing inclusion of fixed effect terms. Best fit model in bold.

| **ASQ domain** | **Model n: Fixed effects terms** | **df** | **AIC** | **BIC** | **ICC** | **Likelihood ratio test (Model n vs n+1)** | | |
| --- | --- | --- | --- | --- | --- | --- | --- | --- |
|  |  |  |  |  |  | $\boldsymbol{\chi}^{\boldsymbol{2}}$ | **p** |  |
| Any Domain | Model 1: Year + Year^2^ + IncDep + Year*IncDep + Year^2^*IncDep | 10 | 178.79 | 221.56 | 0.63 | 0.02 | 0.885 |  |
|  | Model 2: Year + Year^2^ + IncDep + Year*IncDep | 9 | 176.81 | 215.30 | 0.63 | 0.41 | 0.523 |  |
|  | **Model 3: Year + Year^2^ + IncDep** | 8 | 175.22 | 209.43 | 0.63 | 7.93 | **0.005** |  |
|  | Model 4: Year + IncDep | 7 | 181.15 | 211.09 | 0.63 |  |  |  |
| Communication | Model 1: Year + Year^2^ + IncDep + Year*IncDep + Year^2^*IncDep | 10 | 340.77 | 383.58 | 0.46 | 0.01 | 0.942 |  |
|  | Model 2: Year + Year^2^ + IncDep + Year*IncDep | 9 | 338.78 | 377.30 | 0.46 | 0.21 | 0.647 |  |
|  | **Model 3: Year + Year^2^ + IncDep** | 8 | 336.99 | 371.23 | 0.46 | 34.00 | **<0.001** |  |
|  | Model 4: Year + IncDep | 7 | 368.99 | 398.95 | 0.45 |  |  |  |
| Gross Motor | Model 1: Year + Year^2^ + IncDep + Year*IncDep + Year^2^*IncDep | 10 | 791.72 | 834.53 | 0.62 | 0.02 | 0.891 |  |
|  | Model 2: Year + Year^2^ + IncDep + Year*IncDep | 9 | 789.74 | 828.27 | 0.62 | 0.04 | 0.846 |  |
|  | Model 3: Year + Year^2^ + IncDep | 8 | 787.78 | 822.02 | 0.62 | 0.01 | 0.914 |  |
|  | **Model 4: Year + IncDep** | 7 | 785.79 | 815.76 | 0.62 |  |  |  |
| Fine Motor | Model 1: Year + Year^2^ + IncDep + Year*IncDep + Year^2^*IncDep | 10 | 949.62 | 992.43 | 0.65 | 0.40 | 0.527 |  |
|  | Model 2: Year + Year^2^ + IncDep + Year*IncDep | 9 | 948.02 | 986.55 | 0.65 | 0.00 | 0.954 |  |
|  | Model 3: Year + Year^2^ + IncDep | 8 | 946.03 | 980.27 | 0.65 | 2.04 | 0.153 |  |
|  | **Model 4: Year + IncDep** | 7 | 946.07 | 976.03 | 0.65 |  |  |  |
| Problem Solving | Model 1: Year + Year^2^ + IncDep + Year*IncDep + Year^2^*IncDep | 10 | 680.60 | 723.40 | 0.50 | 0.22 | 0.639 |  |
|  | Model 2: Year + Year^2^ + IncDep + Year*IncDep | 9 | 678.81 | 717.34 | 0.50 | 0.71 | 0.401 |  |
|  | **Model 3: Year + Year^2^ + IncDep** | 8 | 677.52 | 711.76 | 0.50 | 12.57 | **<0.001** |  |
|  | Model 4: Year + IncDep | 7 | 688.09 | 718.06 | 0.50 |  |  |  |
| Personal Social Skills | Model 1: Year + Year^2^ + IncDep + Year*IncDep + Year^2^*IncDep | 10 | 586.45 | 629.25 | 0.55 | 0.28 | 0.595 |  |
|  | Model 2: Year + Year^2^ + IncDep + Year*IncDep | 9 | 584.73 | 623.25 | 0.55 | 0.33 | 0.563 |  |
|  | **Model 3: Year + Year^2^ + IncDep** | 8 | 583.06 | 617.31 | 0.55 | 14.62 | **<0.001** |  |
|  | Model 4: Year + IncDep | 7 | 595.69 | 625.65 | 0.56 |  |  |  |

*Note 1: All models were fitted with random intercepts of local authority and random linear slope of year.
Abbreviation: Income deprivation (IncDep)*

eTable 5. Models including adjustment for employment rates at birth

|  | **Any domain** | **Communication** | **Gross Motor** | **Fine Motor** | **Problem Solving** | **Personal social skills** |
| --- | --- | --- | --- | --- | --- | --- |
| *Predictors* | *Estimates* | *Estimates* | *Estimates* | *Estimates* | *Estimates* | *Estimates* |
| (Intercept) | 14.17 ^***^ | 9.35 ^***^ | 3.45 ^***^ | 3.95 ^***^ | 3.74 ^***^ | 5.20 ^***^ |
|  | (10.08 – 19.94) | (6.59 – 13.26) | (1.97 – 6.06) | (1.99 – 7.83) | (2.29 – 6.11) | (3.28 – 8.23) |
| Year | 1.09 ^***^ | 1.20 ^***^ | 1.03 ^*^ | 1.04 ^*^ | 1.20 ^***^ | 1.19 ^***^ |
|  | (1.05 – 1.13) | (1.15 – 1.26) | (1.00 – 1.05) | (1.01 – 1.07) | (1.13 – 1.27) | (1.13 – 1.26) |
| Year^2^ | 0.99 ^**^ | 0.98 ^***^ |  |  | 0.98 ^***^ | 0.98 ^***^ |
|  | (0.99 – 1.00) | (0.97 – 0.99) |  |  | (0.97 – 0.99) | (0.98 – 0.99) |
| Income deprivation | 1.43 ^**^ | 1.53 ^***^ | 1.13 | 1.63 ^*^ | 1.43 ^*^ | 1.40 ^*^ |
|  | (1.16 – 1.76) | (1.25 – 1.88) | (0.81 – 1.59) | (1.07 – 2.49) | (1.07 – 1.91) | (1.06 – 1.83) |
| white ethnicity percent | 1.00 | 1.00 ^**^ | 1.00 | 1.00 | 1.00 | 1.00 |
|  | (0.99 – 1.00) | (0.99 – 1.00) | (1.00 – 1.01) | (0.99 – 1.01) | (1.00 – 1.00) | (0.99 – 1.00) |
| employment percent lag | 1.00 | 1.00 | 1.00 | 1.00 | 1.00 | 1.00 |
|  | (1.00 – 1.00) | (0.99 – 1.00) | (0.99 – 1.01) | (0.99 – 1.00) | (0.99 – 1.00) | (0.99 – 1.00) |
| **Random Effects** |  |  |  |  |  |  |
| σ^2^ | 0.04 | 0.06 | 0.14 | 0.18 | 0.13 | 0.09 |
| τ_00_ | 0.12 _AreaName_ | 0.12 _AreaName_ | 0.25 _AreaName_ | 0.42 _AreaName_ | 0.20 _AreaName_ | 0.23 _AreaName_ |
| τ_11_ | 0.00 _AreaName.I(YearN)_ | 0.00 _AreaName.I(YearN)_ | 0.01 _AreaName.I(YearN)_ | 0.01 _AreaName.I(YearN)_ | 0.00 _AreaName.I(YearN)_ | 0.01 _AreaName.I(YearN)_ |
| ρ_01_ | -0.53 _AreaName_ | -0.66 _AreaName_ | -0.44 _AreaName_ | -0.45 _AreaName_ | -0.56 _AreaName_ | -0.67 _AreaName_ |
| ICC | 0.71 | 0.56 | 0.62 | 0.68 | 0.56 | 0.62 |
| N | 118 _AreaName_ | 118 _AreaName_ | 118 _AreaName_ | 118 _AreaName_ | 118 _AreaName_ | 118 _AreaName_ |
| Observations | 531 | 533 | 533 | 533 | 533 | 533 |
| Marginal R^2^ / Conditional R^2^ | 0.138 / 0.753 | 0.233 / 0.662 | 0.016 / 0.628 | 0.056 / 0.697 | 0.124 / 0.614 | 0.137 / 0.676 |
| AIC | 223.45 | 376.85 | 815.79 | 974.41 | 718.26 | 623.75 |

*Note: * p<0.05   ** p<0.01   *** p<0.001*

eTable 6. Number (%) of local authorities with published data on the Ages and Stages Qusetionnaire 3 2018 to 2024

| **Year** | **Any domain** | **Communication** | **Gross Motor** | **Fine Motor** | **Problem Solving** | **Personal social skills** |
| --- | --- | --- | --- | --- | --- | --- |
| 2018 | 74 (50.0%) | 75 (50.7%) | 75 (50.7%) | 75 (50.7%) | 75 (50.7%) | 74 (50%) |
| 2019 | 113 (76.4%) | 116 (78.4%) | 115 (77.7%) | 115 (77.7%) | 115 (77.7%) | 114 (77%) |
| 2020 | 129 (87.2%) | 127 (85.8%) | 127 (85.8%) | 127 (85.8%) | 127 (85.8%) | 126 (85.1%) |
| 2021 | 134 (90.5%) | 135 (91.2%) | 134 (90.5%) | 134 (90.5%) | 134 (90.5%) | 133 (89.9%) |
| 2022 | 138 (93.2%) | 138 (93.2%) | 138 (93.2%) | 138 (93.2%) | 137 (92.6%) | 137 (92.6%) |
| 2023 | 132 (89.2%) | 132 (89.2%) | 132 (89.2%) | 132 (89.2%) | 132 (89.2%) | 131 (88.5%) |
| 2024 | 137 (92.6%) | 137 (92.6%) | 137 (92.6%) | 137 (92.6%) | 137 (92.6%) | 136 (91.9%) |
| 2025 | 139 (93.9%) | 139 (93.9%) | 139 (93.9%) | 139 (93.9%) | 139 (93.9%) | 139 (93.9%) |

eTable 7. Correlation between coverage of developmental review using the Ages and Stages Questionnaire and income deprivation score

| **Year** | **Kendall tau** | **p** |
| --- | --- | --- |
| 2018 | 0.141 | 0.015 |
| 2019 | 0.022 | 0.701 |
| 2020 | 0.102 | 0.074 |
| 2021 | 0.126 | 0.029 |
| 2022 | 0.120 | 0.034 |
| 2023 | 0.070 | 0.229 |
| 2024 | 0.089 | 0.118 |
| 2025 | 0.088 | 0.119 |

eTable 8. Coverage of developmental review using the Ages and Stages Questionnaire, amongst local authorities reporting data on coverage (x)

|  | **Number of local authorities with percent coverage (x) in the following ranges** | | | | | **Total local authorities reporting coverage** |
| --- | --- | --- | --- | --- | --- | --- |
| **Year** | **0% ≤ x < 25%** | **25% ≤ x < 50%** | **50% ≤ x < 75%** | **75% ≤ x ≤ 100%** | **>100%** |  |
| 2018 | 2 | 12 | 55 | 69 | 0 | 138 |
| 2019 | 0 | 6 | 54 | 83 | 1 | 143 |
| 2020 | 2 | 5 | 44 | 88 | 2 | 139 |
| 2021 | 10 | 21 | 56 | 47 | 4 | 134 |
| 2022 | 5 | 15 | 50 | 70 | 2 | 140 |
| 2023 | 7 | 11 | 42 | 72 | 3 | 132 |
| 2024 | 3 | 10 | 30 | 93 | 6 | 136 |
| 2025 | 0 | 2 | 36 | 101 | 4 | 139 |

*Note: greater than 100% coverage results because the recorded number of children receiving an ASQ review exceeds the recorded number of children eligible for a developmental review*

eTable 9. Analysed data – number (%) of local authorities with published data on ASQ based on coverage more than 75% and data on income deprivation after excluding poor quality data and extreme outliers.

| **Year** | **Any domain** | **Communication** | **Gross Motor** | **Fine Motor** | **Problem Solving** | **Personal social skills** |
| --- | --- | --- | --- | --- | --- | --- |
| 2018 | NA | NA | NA | NA | NA | NA |
| 2019 | 69 (46.6%) | 71 (48%) | 71 (48%) | 71 (48%) | 71 (48%) | 71 (48%) |
| 2020 | 81 (54.7%) | 81 (54.7%) | 81 (54.7%) | 81 (54.7%) | 81 (54.7%) | 81 (54.7%) |
| 2021 | 46 (31.1%) | 46 (31.1%) | 46 (31.1%) | 46 (31.1%) | 46 (31.1%) | 46 (31.1%) |
| 2022 | 69 (46.6%) | 69 (46.6%) | 69 (46.6%) | 69 (46.6%) | 69 (46.6%) | 69 (46.6%) |
| 2023 | 74 (50%) | 74 (50%) | 74 (50%) | 74 (50%) | 74 (50%) | 74 (50%) |
| 2024 | 95 (64.2%) | 95 (64.2%) | 95 (64.2%) | 95 (64.2%) | 95 (64.2%) | 95 (64.2%) |
| 2025 | 98 (66.2%) | 98 (66.2%) | 98 (66.2%) | 98 (66.2%) | 98 (66.2%) | 98 (66.2%) |

eTable 10. Difference in predicted mean rates of children not on track in development, relative to 2019, with bootstrapped confidence intervals in brackets

|  | **Any domain** | **Communication** | **Gross Motor** | **Fine Motor** | **Problem Solving** | **Personal Social Skills** |  |
| --- | --- | --- | --- | --- | --- | --- | --- |
| ***Absolute difference*** | | | | | | | |
| 2019 | Ref | | | | | | |
| 2020 | 1.1 (0.6 to 1.6) | 1.4 (1 to 1.8) | 0.1 (0 to 0.2) | 0.1 (0 to 0.2) | 0.7 (0.4 to 0.9) | 0.8 (0.5 to 1.1) |  |
| 2021 | 2.1 (1.2 to 2.9) | 2.6 (1.9 to 3.3) | 0.3 (0.1 to 0.4) | 0.3 (0.1 to 0.4) | 1.4 (0.8 to 1.8) | 1.5 (1.0 to 2.0) |  |
| 2022 | 2.8 (1.7 to 3.8) | 3.5 (2.6 to 4.4) | 0.4 (0.1 to 0.7) | 0.4 (0.1 to 0.7) | 1.9 (1.1 to 2.4) | 2.1 (1.4 to 2.8) |  |
| 2023 | 3.3 (2.1 to 4.4) | 4 (3.0 to 4.9) | 0.6 (0.2 to 0.9) | 0.6 (0.2 to 0.9) | 2.2 (1.4 to 2.8) | 2.6 (1.8 to 3.2) |  |
| 2024 | 3.6 (2.4 to 4.5) | 3.9 (3 to 4.7) | 0.7 (0.2 to 1.2) | 0.7 (0.2 to 1.2) | 2.4 (1.7 to 2.9) | 2.7 (2.1 to 3.3) |  |
| 2025 | 3.6 (2.5 to 4.4) | 3.4 (2.7 to 4.1) | 0.9 (0.3 to 1.4) | 0.9 (0.2 to 1.4) | 2.3 (1.8 to 2.9) | 2.7 (2.1 to 3.2) |  |
| ***Relative difference*** | | | | | | | |
| 2019 | Ref | | | | | | |
| 2020 | 1.08  (1.04 to 1.12) | 1.18  (1.12 to 1.24) | 1.03  (1.01 to 1.05) | 1.03  (1.01 to 1.06) | 1.17  (1.08 to 1.25) | 1.17  (1.09 to 1.23) |  |
| 2021 | 1.15  (1.08 to 1.22) | 1.33  (1.22 to 1.45) | 1.06  (1.02 to 1.1) | 1.07  (1.02 to 1.11) | 1.33  (1.17 to 1.48) | 1.32  (1.18 to 1.45) |  |
| 2022 | 1.21  (1.11 to 1.29) | 1.44  (1.29 to 1.59) | 1.09  (1.03 to 1.15) | 1.1  (1.03 to 1.18) | 1.46  (1.24 to 1.66) | 1.44  (1.26 to 1.61) |  |
| 2023 | 1.24  (1.14 to 1.33) | 1.49  (1.34 to 1.66) | 1.12  (1.04 to 1.21) | 1.14  (1.03 to 1.24) | 1.55  (1.32 to 1.76) | 1.53  (1.33 to 1.72) |  |
| 2024 | 1.26  (1.16 to 1.35) | 1.49  (1.35 to 1.63) | 1.15  (1.04 to 1.27) | 1.17  (1.04 to 1.31) | 1.59  (1.37 to 1.8) | 1.56  (1.39 to 1.74) |  |
| 2025 | 1.26  (1.17 to 1.34) | 1.42  (1.30 to 1.54) | 1.19  (1.05 to 1.33) | 1.21  (1.05 to 1.38) | 1.58  (1.40 to 1.77) | 1.55  (1.40 to 1.70) |  |

eTable 11. Sensitivity analysis – models of year and income deprivation on early childhood development, using data based on at least 50% ASQ coverage, with adjustment for ASQ coverage

|  | **Any domain** | **Communication** | **Gross Motor** | **Fine Motor** | **Problem Solving** | **Personal social skills** |
| --- | --- | --- | --- | --- | --- | --- |
| *Predictors* | *Estimates* | *Estimates* | *Estimates* | *Estimates* | *Estimates* | *Estimates* |
| (Intercept) | 18.57 ^***^ | 12.68 ^***^ | 5.04 ^***^ | 5.38 ^***^ | 5.94 ^***^ | 7.07 ^***^ |
|  | (14.12 – 24.43) | (9.55 – 16.85) | (3.25 – 7.80) | (3.14 – 9.20) | (4.05 – 8.69) | (4.95 – 10.12) |
| Year | 1.09 ^***^ | 1.16 ^***^ | 1.03 ^*^ | 1.03 ^**^ | 1.07 ^***^ | 1.16 ^***^ |
|  | (1.06 – 1.13) | (1.12 – 1.20) | (1.00 – 1.05) | (1.01 – 1.06) | (1.05 – 1.09) | (1.11 – 1.21) |
| Year^2^ | 0.99 ^***^ | 0.98 ^***^ |  |  |  | 0.99 ^***^ |
|  | (0.99 – 1.00) | (0.98 – 0.99) |  |  |  | (0.98 – 0.99) |
| Income Deprivation | 1.34 ^**^ | 1.49 ^***^ | 1.17 | 1.67 ^**^ | 1.57 ^***^ | 1.48 ^**^ |
|  | (1.11 – 1.63) | (1.23 – 1.79) | (0.86 – 1.58) | (1.15 – 2.42) | (1.22 – 2.03) | (1.17 – 1.87) |
| White ethnicity percent | 1.00 ^*^ | 1.00 ^**^ | 1 | 1 | 1 | 1 |
|  | (0.99 – 1.00) | (0.99 – 1.00) | (1.00 – 1.01) | (0.99 – 1.00) | (1.00 – 1.00) | (1.00 – 1.00) |
| ASQ coverage | 1.00 ^*^ | 1.00 ^*^ | 1.00 ^*^ | 0.99 ^*^ | 0.99 ^***^ | 0.99 ^***^ |
|  | (1.00 – 1.00) | (0.99 – 1.00) | (0.99 – 1.00) | (0.99 – 1.00) | (0.99 – 1.00) | (0.99 – 1.00) |
| **Random Effects** |  |  |  |  |  |  |
| σ^2^ | 0 | 0.1 | 0.2 | 0.2 | 0.1 | 0.1 |
| τ_00_ | 0.1 _AreaName_ | 0.1 _AreaName_ | 0.4 _AreaName_ | 0.5 _AreaName_ | 0.3 _AreaName_ | 0.3 _AreaName_ |
| τ_11_ | 0.0 _AreaName.I(YearN)_ | 0.0 _AreaName.I(YearN)_ | 0.0 _AreaName.I(YearN)_ | 0.0 _AreaName.I(YearN)_ | 0.0 _AreaName.I(YearN)_ | 0.0 _AreaName.I(YearN)_ |
| ρ_01_ | -0.6 _AreaName_ | -0.7 _AreaName_ | -0.6 _AreaName_ | -0.5 _AreaName_ | -0.7 _AreaName_ | -0.7 _AreaName_ |
| ICC | 0.7 | 0.6 | 0.6 | 0.7 | 0.6 | 0.6 |
| N | 142 _AreaName_ | 142 _AreaName_ | 142 _AreaName_ | 142 _AreaName_ | 142 _AreaName_ | 142 _AreaName_ |
| Observations | 790 | 792 | 792 | 792 | 791 | 792 |
| Marginal R^2^ / Conditional R^2^ | 0.119 / 0.747 | 0.187 / 0.673 | 0.014 / 0.640 | 0.046 / 0.676 | 0.097 / 0.646 | 0.109 / 0.676 |
| AIC | 370.53 | 604.83 | 1226.88 | 1502.26 | 1063.43 | 926.82 |

eTable 12. Summary of cost estimates based on the 2025 increase in children not on track in development, compared to pre-pandemic.

| **Cost Category** | **Cost assumptions (Source)** | **Calculation of cost incurred** | **Cost incurred^*^** |
| --- | --- | --- | --- |
| Contemporary costs | | | |
| Increased early intervention (speech and language therapy) | £2,000 per affected child annually  (NHS England, 2023)^7^ | (540,000*0.04*0.1*£2,000) | £4.32 million annually |
| Increased demand for Special Education Needs support | £10,000 per affected child annually  (Education Policy Institute, 2020)^8^ | (540,000*0.04*0.1*£10,000) | £21.6 million annually |
| Parental employment losses | Average employment loss of £7,500 per affected child annually (OECD, 2021)^9^ | (540,000*0.04*0.1*£7,500) | £16.2 million annually |
| Predicted future costs | | | |
| Lower workforce participation resulting from lower educational attainment | £100,000 reduction in lifetime earnings per child who have lower educational attainment  (UK Department for Education, 2021)^5^ | (540,000*0.04*0.1*£100,000) | £216 million in lifetime earnings loss |
| Reduced economic productivity | 10% reduction in productivity amongst children affected  (Penn Wharton Budget Model, 2021)^6^ | (0.04*0.1*10%*UK GDP^^^) | Approximately £1 billion fall in UK GDP^^^ |

*^*^Costs incurred are calculated based on published costs, the 2025 estimate that around 4 per 100 more children fail the ASQ-3 developmental screen at 2-2.5 years, and that 10% of children failing the early developmental screen require additional services/greater parental support, and experience lower educational attainment/workforce participation and reduced economic productivity in adulthood (ie., 90% of children affected by COVID-19 catch up in their development over time).*^2,3^

*^^^ USD 3.38 trillion (approximately £2.6 trillion) in 2023.*^10^

*Note: cited references are listed in the reference section of Supplement 1*

eFigure 1. Time trends in the coverage of Ages and Stages Questionnaire 3 developmental review by IMD quintile, all published data on children assessed

eFigure 2. Histogram showing variation in coverage of Ages and Stages Questionnaire 3 developmental review, all published data on children assessed

eFigure 3. Raincloud plot (top) and normal q-q plot (bottom) showing the positive skew of the outcome in the original scale, analysis sample

eFigure 4. Raincloud plot (top) and normal q-q plot (bottom) of the log-transformed outcome showing it approximates a normal distribution, analysis sample

eFigure 5. Residual diagnostics for models using the log-transformed outcome. No trend in the residuals against fitted values or the variance in the residuals across different fitted values

eFigure 6. Residual diagnostics for models using the log-transformed outcome. Residuals are normally distributed

eFigure 7. Model diagnostics. Random effects are correctly specified (intercepts for each local authority follows a normal distribution

eFigure 8. Inclusion and exclusion criteria for analysis
